# Genipin-Induced Inhibition of Uncoupling Protein-2 Sensitizes Drug-Resistant Cancer Cells to Cytotoxic Agents

**DOI:** 10.1371/journal.pone.0013289

**Published:** 2010-10-13

**Authors:** Ryan J. Mailloux, Cyril Nii-Klu Adjeitey, Mary-Ellen Harper

**Affiliations:** Department of Biochemistry, Microbiology and Immunology, Faculty of Medicine, University of Ottawa, Ottawa, Canada; Hospital 12 Octubre Madrid, Spain

## Abstract

Uncoupling protein-2 (UCP2) is known to suppress mitochondrial reactive oxygen species (ROS) production and is employed by drug-resistant cancer cells to mitigate oxidative stress. Using the drug-sensitive HL-60 cells and the drug-resistant MX2 subline as model systems, we show that genipin, a UCP2 inhibitor, sensitizes drug-resistant cells to cytotoxic agents. Increased MX2 cell death was observed upon co-treatment with genipin and different doses of menadione, doxorubicin, and epirubicin. DCFH-DA fluorimetry revealed that the increase in MX2 cell death was accompanied by enhanced cellular ROS levels. The drug-induced increase in ROS was linked to genipin-mediated inhibition of mitochondrial proton leak. State 4 and resting cellular respiratory rates were higher in the MX2 cells in comparison to the HL-60 cells, and the increased respiration was readily suppressed by genipin in the MX2 cells. UCP2 accounted for a remarkable 37% of the resting cellular oxygen consumption indicating that the MX2 cells are functionally reliant on this protein. Higher amounts of UCP2 protein were detected in the MX2 *versus* the HL-60 mitochondria. The observed effects of genipin were absent in the HL-60 cells pointing to the selectivity of this natural product for drug-resistant cells. The specificity of genipin for UCP2 was confirmed using CHO cells stably expressing UCP2 in which genipin induced an ∼22% decrease in state 4 respiration. These effects were absent in empty vector CHO cells expressing no UCP2. Thus, the chemical inhibition of UCP2 with genipin sensitizes multidrug-resistant cancer cells to cytotoxic agents.

## Introduction

Intrinsic or acquired drug resistance to chemotherapeutic agents represents a great obstacle facing the successful eradication of cancers [1]. The ability of cancer cells to evade drug toxicity is attributed to the induction of elaborate detoxification mechanisms. Indeed, the chronic exposure of cancer cells to chemotherapeutic agents can elicit pro-survival responses characterized by the enhanced ability to render chemotherapeutics innocuous [2], [3]. Although cancer cells invoke numerous strategies to nullify toxins, tight control over ROS levels represents a major adaptive response [4]. Ensuring that ROS levels remain in the non-toxic range is a continuous challenge for cancer cells Indeed, cancer cells are continually exposed to high levels of ROS produced by compromised aerobic metabolism, chemotherapeutics, nutrient deprivation, and host immune responses [5], [6], [7]. If left unchecked these singlet electron carriers can damage essential cellular macromolecules leading to cell death [8], [9]. Hence, a suite of anti-oxidative defense strategies are invoked by cancer cells to maintain ROS levels within tolerable limits.

The induction of UCP2 represents one of the many adaptive mechanisms invoked by drug-resistant cells to maintain ROS homeostasis [10], [11]. UCP2 belongs to the mitochondrial anion carrier superfamily and holds high homology to the original mitochondrial uncoupling protein that is highly selectively expressed in brown fat, UCP1 [12]. Various studies have shown that UCP2 can prevent oxidative stress by increasing the flow of protons into the matrix thus rendering electron flow through the respiratory complexes more efficient [13], [14], [15]. This function of UCP2 may be especially important in cancer cells since their mitochondria are metabolically abnormal [16].

ROS are an inherent by-product of aerobic respiration since mitochondrial respiratory complexes I and III can partake in the singlet electron reduction of diatomic oxygen [17]. Roughly 0.2–2% of the O_2_ metabolized by the mitochondria is converted to ROS [18]. However, ROS generation can be greatly enhanced by conditions that over-supply reducing equivalents to the respiratory complexes thereby increasing mitochondrial membrane potential (*Δψ*_m_). In fact, it has been reported in several studies that ROS formation by the respiratory chain is strongly dependent upon *Δψ*_m_ [19], [20]. Thus, even slight depolarization of the *Δψ*_m_ can enhance electron flow through the complexes and diminish mitochondrial ROS production. UCP2 may fulfill this uncoupling role in cancer cells; other reports describe the expression of other uncoupling proteins, including UCP3 and UCP5 in adenocarcinoma and colon cancer cells [21], [22]. Indeed, various *in vivo* and *in vitro* studies have shown that UCP2 is a *bona fide* uncoupler of oxidative phosphorylation and limits oxidative injury to tissues while loss of UCP2 function increases mitochondrial ROS production [13], [23], [24], [25], [26]. Furthermore, the uncoupling activity of UCP2 is thought to provide a negative feedback loop for the acute control of ROS formation by mitochondria [27]. Thus, UCP2 may play an integral role in the adaptive response of cancer cells to chemotherapeutics.

Inhibiting drug-resistance mechanisms represents one method for sensitizing cancer cells to chemotherapeutics [21], [22]. UCP2 overexpression has been described in various types of cancer including leukemia cells, human colon cancer cells, thyroid tumours, and hepatomas [28], [29], [30], [31]. Very recently, UCP2 knock-down has also been shown to enhance the toxic effects of the chemotherapeutic cisplatin [32]. More intriguingly, UCP2 overexpression has been suggested to be part of the adaptive mechanism required for the survival of cancer cells in adverse environments [11]. For instance, overexpression of UCP2 in HCT116 human colon cancer cells diminishes the pro-apoptotic effects of the chemotherapeutics, etoposide and doxorubicin [33]. This was also observed with drug-resistant L1210/DDP leukemia cells which use UCP2 to quench chemotherapy-induced ROS through a proton leak mechanism [10]. Thus, UCP2 may represent a unique target for the sensitization of drug-resistant cells to chemotherapeutics. The aim of the current study was to test the hypothesis that genipin, an extract from *Gardenia jasminoides* which has been shown to inhibit UCP2 and improve insulin secretion, can inhibit UCP2 in the drug resistant MX2 cancer cells to increase oxidative stress and susceptibility to cytotoxic agents. Genipin has been shown to be a highly selective inhibitor of UCP2. Indeed, several studies have provided evidence that genipin specifically impedes UCP2 mediated proton leak in pancreatic β-cells, kidney mitochondria, and in brain tissue [34], [35]. Of note also is the fact that genipin is a traditional Chinese remedy for the treatment of type 2 diabetes mellitus [35].

## Results and Discussion

### Genipin sensitizes drug-resistant leukemia cells to ROS toxicity

**Figure 1:**
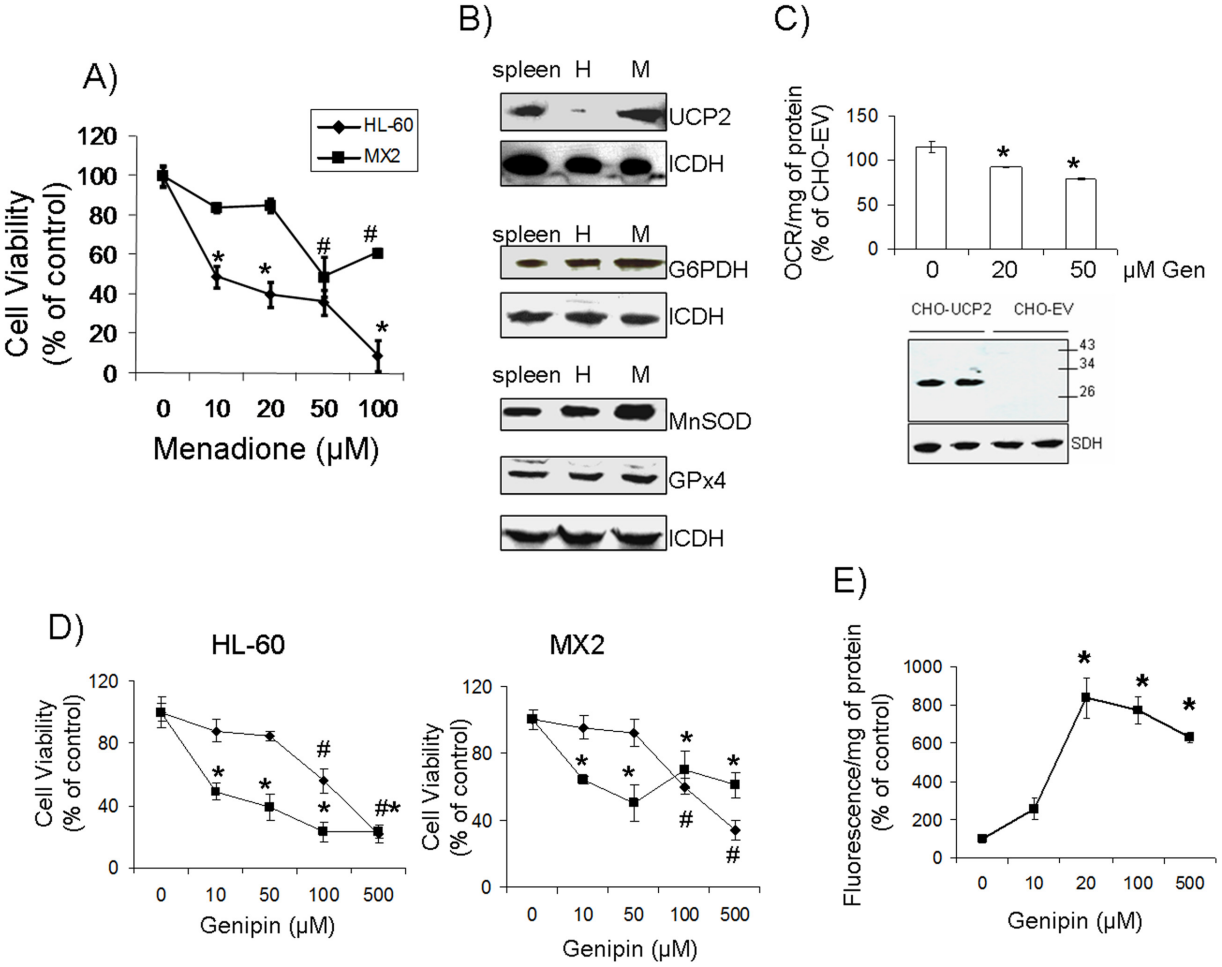
Genipin sensitizes drug-resistant MX2 cells to menadione toxicity. A) The viability of HL-60 (filled diamond) and MX2 (filled square) following exposure to menadione. Cells were exposed for 24 h to different concentrations of menadione (0–100 µmol/L) followed by the assessment of the number of live cells. Viability was expressed as a percent of the control value. Kruskall-Wallis with a post-hoc Mann-Whitney test, n = 3, p<0.05. * indicates statistical significance for HL-60 cells compared to control and # indicates statistical significance for MX2 compared to control. B) Immunoblot analysis of anti-oxidant defense enzymes and UCP2. Mitochondria from MX2 (M) and HL-60 (H) cells were isolated and analyzed for UCP2, MnSOD, and GPx4. Cytosol was used for G6PDH determinations. NADP-ICDH served as the loading control for the cytosol and mitochondrial fractions. Spleen lysate (100 µg) was used as a UCP2 loading control. The molecular weight of UCP2 was confirmed using molecular mass markers. C) *In situ* analysis of the specificity of genipin for UCP2. Following the determination of basal oxygen consumption rates, the impact of genipin on OCR in CHO-UCP2 (white bars) cells was assessed. Cells were exposed to (0–50 µM) genipin for 15 min and then OCR was tested. Data was expressed as a % of CHO-EV OCR. *INSET*: immunoblot analysis of UCP2 in CHO-UCP2 and CHO-EV cells. SDH served as the loading control. Kruskall-Wallis with a post-hoc Mann-Whitney test, n = 3, p<0.05. * indicates statistical significance when compared to the control. D) Assessment of genipin toxicity and the response of drug-sensitive HL-60 and drug-resistant MX2 cells to simultaneous treatment with menadione and genipin. Cells were exposed to genipin (0–500 µmol/L) in the presence or absence of 20 µM menadione for 24 h. Following the exposure, cell viability was determined. ♦ and ▪ represent exposure to either genipin or genipin + menadione. Viability was expressed as a percent of the control. Kruskall-Wallis with a post-hoc Mann-Whitney test, n = 3, p<0.05. * corresponds to statistical significance when cells exposed to both menadione and genipin were compared to control. # corresponds to statistical significance when genipin-exposed cells were compared to the control cells. E) Treatment with genipin and menadione increases MX2 cell death. MX2 cells were exposed to 20 µmol/L menadione and genipin (0–500 µmol/L) for 24 h. The amount of cell death was determined using the PI assay. 1-way ANOVA with a post-hoc Tukey's test, n = 5, p<0.05. * represents significance when compared to 0 µM.

The emergence of drug resistant cancer phenotypes is accompanied by the acquisition of efficient control over ROS homeostasis. UCP2 is an important modulator of ROS production in drug-resistant cancer cells [10], [11]. This mitochondrial inner membrane protein is expressed at high levels in drug-resistant cells and has been shown to play a key role in curtailing oxidative stress [10], [36]. Thus, we sought to determine if chemical inhibition of UCP2 could render drug-resistant cells more sensitive to cytotoxic agents. The drug-resistant MX2 cells displayed increased resistance to the superoxide-producing menaquinone, menadione (**Figure 1A**). Menadione produces ROS by rapidly cycling between the quinone and semiquinone state and is used frequently to mimic oxidative stress [37]. Indeed, HL-60 viability decreased substantially upon treatment with 10 µM menadione. Furthermore, exposure of HL-60 cells to 100 µM menadione resulted in a 90% decrease in cell viability. However, the MX2 cells retained cell viability with concentrations of menadione up to 100 µM (**Figure 1A**). Immunoblot analyses revealed that the mitochondria from the MX2 cells contained higher amounts of UCP2 protein (**Figure 1B**). In contrast to other studies, we found that the anti-N19 antibody provided a very clear chemiluminescent signal [38]. The electrophoretic mobility of UCP2 was confirmed using spleen lysate. The anti-C20 antibody did not provide a signal even with the spleen lysate (data not shown). Immunoblot analyses revealed only slight increases in other anti-oxidative defense enzymes in the MX2 cells (**Figure 1B**). Hence, although the drug-resistant MX2 cells increase several anti-oxidative defense enzymes to increase ROS handling, UCP2 displays the greatest increase in expression when compared to the drug-sensitive HL-60 counterpart. The specificity of genipin for UCP2 was confirmed using CHO cells stably transfected with either UCP2 or empty vector control. Immunoblotting confirmed that UCP2 is expressed only in the CHO-UCP2 cells (**Figure 1C**). The single band in each lane of the immunoblots also aligned at ∼32 KDa indicating the specificity of the antibody for UCP2 protein. *In situ* bioenergetic determinations on CHO cells stably transfected with UCP2 or empty vector were used to test the specificity of genipin. Measurement of basal OCR prior to genipin treatment revealed that the CHO-UCP2 cells displayed a significantly higher oxygen consumption rate that was ∼18% more elevated than the CHO-EV cells (852.523±45.838 OCR/mg of protein CHO-UCP3 vs 721.352±15.050 OCR/mg of protein CHO-EV, p<0.05, n = 3). However, genipin treatment (20 and 50 µM) lead to an 12 and 22% decrease in the OCR of the CHO-UCP2 cells in comparison to the CHO-EV cells (**Figure 1C**). Hence, these data clearly illustrate the specific inhibitory action of genipin on UCP2. It is worthy to note that genipin treatment had no effect on the OCR of the CHO-EV cells illustrating the specificity of genipin for UCP2.

It is clear from the above data that MX2 cells express high amounts UCP2 and genipin specifically inhibits UCP2. Next we evaluated if genipin could sensitize the MX2 cells to menadione toxicity. As well as its use in treating type 2 diabetes, genipin has been employed to treat glioma, hepatomas, and has anti-inflammatory properties [35], [39], [40], [41]. However, a dearth of information exists on genipin toxicity. Our results show that genipin concentrations higher than 50 µM resulted in the loss of cell viability in both HL-60 and MX2 cultures (**Figure 1D**). This is most likely due to its protein crosslinking abilities. Genipin crosslinking results in the production of a blue pigment. However, no blue pigment was observed at concentrations of genipin <100 µM (data not shown). Concentrations below 50 µM had no significant impact on the viability of either the MX2 or HL-60 cells. More importantly, exposure of MX2 cells to a combination of 20 µM menadione and genipin (concentration as low as 10 µM) resulted in a sharp decline in cell viability (**Figure 1D**). There was an increase in MX2 viability upon exposure to <100 µM genipin in combination with menadione however there was still a significant decrease in viability when compared to control. The sensitizing effect of genipin to menadione was confirmed using the PI assay. Exposure of MX2 cells for 24 h to 20 µM menadione in the presence of 10 or 20 µM genipin resulted in a 2-fold and 8-fold increase in cell death, respectively (**Figure 1E**). In contrast, concentrations of menadione ≥50 µM were required to induce MX2 death when genipin was absent (**Figure S1**). In addition, the increase in cell death was several-fold higher when genipin was included. Propidium iodide uptake can occur in both necrotic and apoptotic cells. Thus, we measured several apoptosis markers. No active caspase-3 was detected and cytochrome C was absent from the cytosol consistent with the induction of necrosis (data not shown). Although these two apoptosis biomarkers were not detected, a more in-depth analysis of the mechanism of cell death is required. We also assessed the impact of different amounts of genipin on the non-cancerous C2C12 myoblast cell line. C2C12 myoblasts displayed an increase in cell death when treated with ≥50 µM genipin (**Figure S1**). These observations indicate that genipin does not induce a significant loss in cell viability at concentrations below 50 µM and the effects of genipin on the MX2 cells are due to its interactions with UCP2. Despite these observations, more *in vivo* analyses on the toxicity of genipin and its applicability to the treatment of cancer in the clinical setting are required. Thus, genipin sensitizes drug-resistant cells expressing UCP2 to ROS-producing agents.

### Genipin sensitizes MX2 cells to anthracyclin toxicity

**Figure 2:**
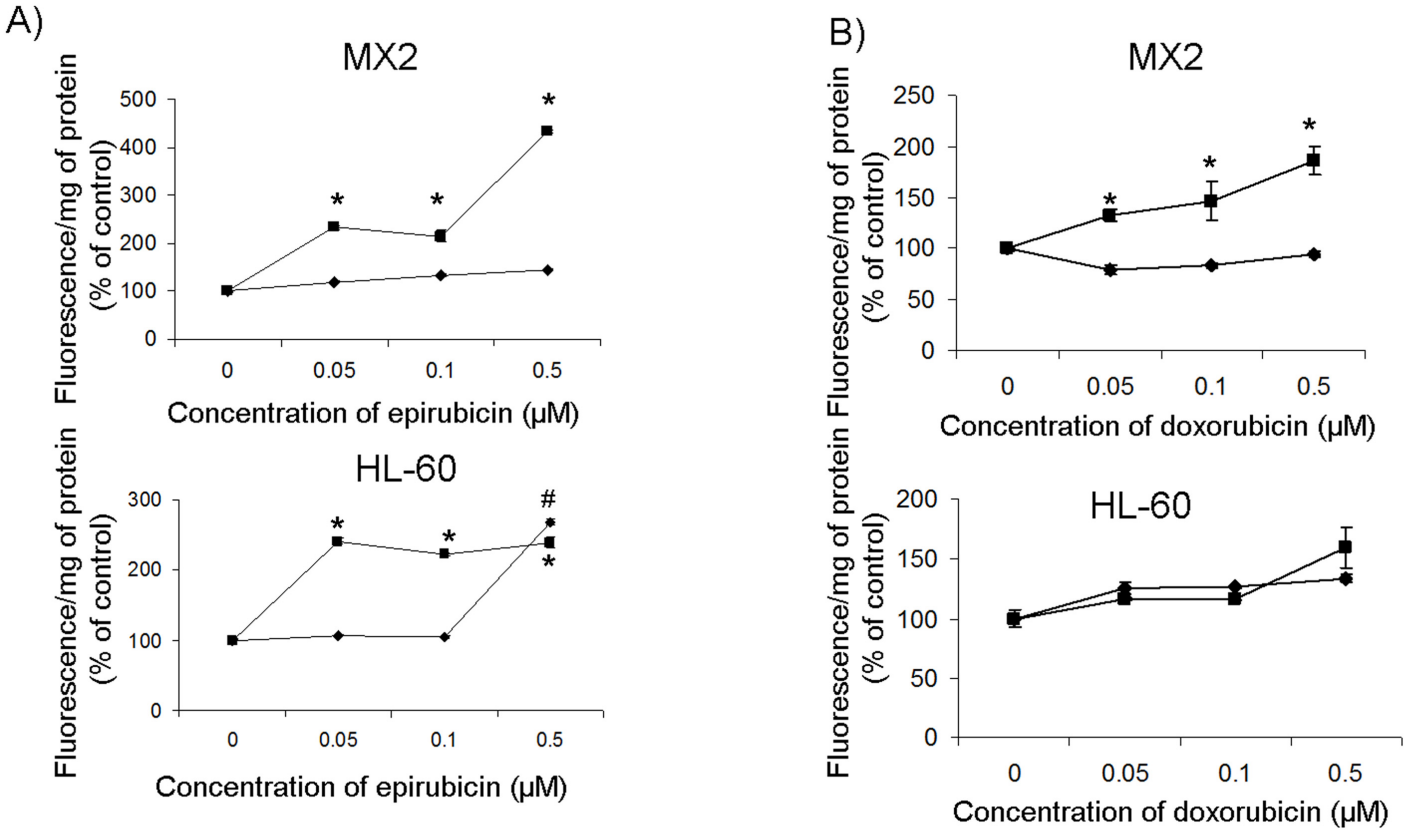
Genipin sensitizes MX2 cells to epirubicin and doxorubicin. HL-60 and MX2 cells were grown to 70% confluency and then exposed to either epirubicin (0–0.5 µM) or doxorubicin (0–0.5 µM) in the absence or presence of 20 µM genipin. Amount of cell death was determined by PI assay. Results were expressed as a percent of the control. A) ♦ epirubicin only, ▪ epirubicin + genipin. 1-way ANOVA with a post-hoc Tukey's test, n = 5, * p<0.05. * corresponds to statistical significance when epirubicin and genipin treated cells were compared to control. # corresponds to statistical significance when epirubicin-treated cells were compared to control cells. B) ♦ doxorubicin only, ▪ doxorubicin + genipin. 1-way ANOVA with a post-hoc Tukey's test, n = 5, p<0.05. * corresponds to statistical significance when epirubicin and genipin treated cells were compared to control cells.

Menadione has been shown to have an anti-cancer effect and has been employed with mitomycin C in phase II trials to treat advanced lung cancer [42]. However, menadione is not currently employed as a cancer chemotherapeutic. Thus, we determined if genipin could sensitize the MX2 cells to commonly used chemotherapeutics, specifically the quinone-based anthracyclins, doxorubicin and epirubicin. MX2 cells are well known to be resistant to this class of chemotherapeutics [43]. As shown in **Figure 2A**, the MX2 cells were more resistant to increased epirubicin concentrations (0.5 µM) in contrast to their drug-sensitive counterpart. However, sharp increments in cell death were observed in the genipin-treated MX2 cells exposed to increasing amounts of epirubicin (**Figure 2A**). Curiously, genipin and epirubicin (0.05–0.1 µM) co-treatment increased cell death in the HL-60 cells but this response was nullified at higher doses epirubicin (0.5 µM, MX2 cells displayed a 4-fold increase whereas the HL-60 cells displayed a 2-fold increase). MX2 cells were also resistant to doxorubicin treatment (**Figure 2B**). Intriguingly, the HL-60 cells only displayed a trend toward increased cell death, however statistical significance was not reached (0.05–0.5 µM doxorubicin, only a 20–40% increase; p>0.05). The major observation in **Figure 2B** is the significant increase in MX2 cell death upon treatment with genipin and doxorubicin (0.05–0.5 µM; ∼50–110%; p<0.05). The sensitizing effect of genipin was not observed in the HL-60 cells treated with doxorubicin which is consistent with the inhibitory action of genipin on UCP2. Use of epirubicin and doxorubicin concentrations above 1 µM in combination with genipin resulted in drastic increases in the cell death of HL-60 and MX2 cells (data not shown). Hence, genipin disarms chemotherapeutic resistance in drug-resistant cells overexpressing UCP2.

### Genipin treatment perturbs aerobic respiration in drug-resistant cells

**Figure 3:**
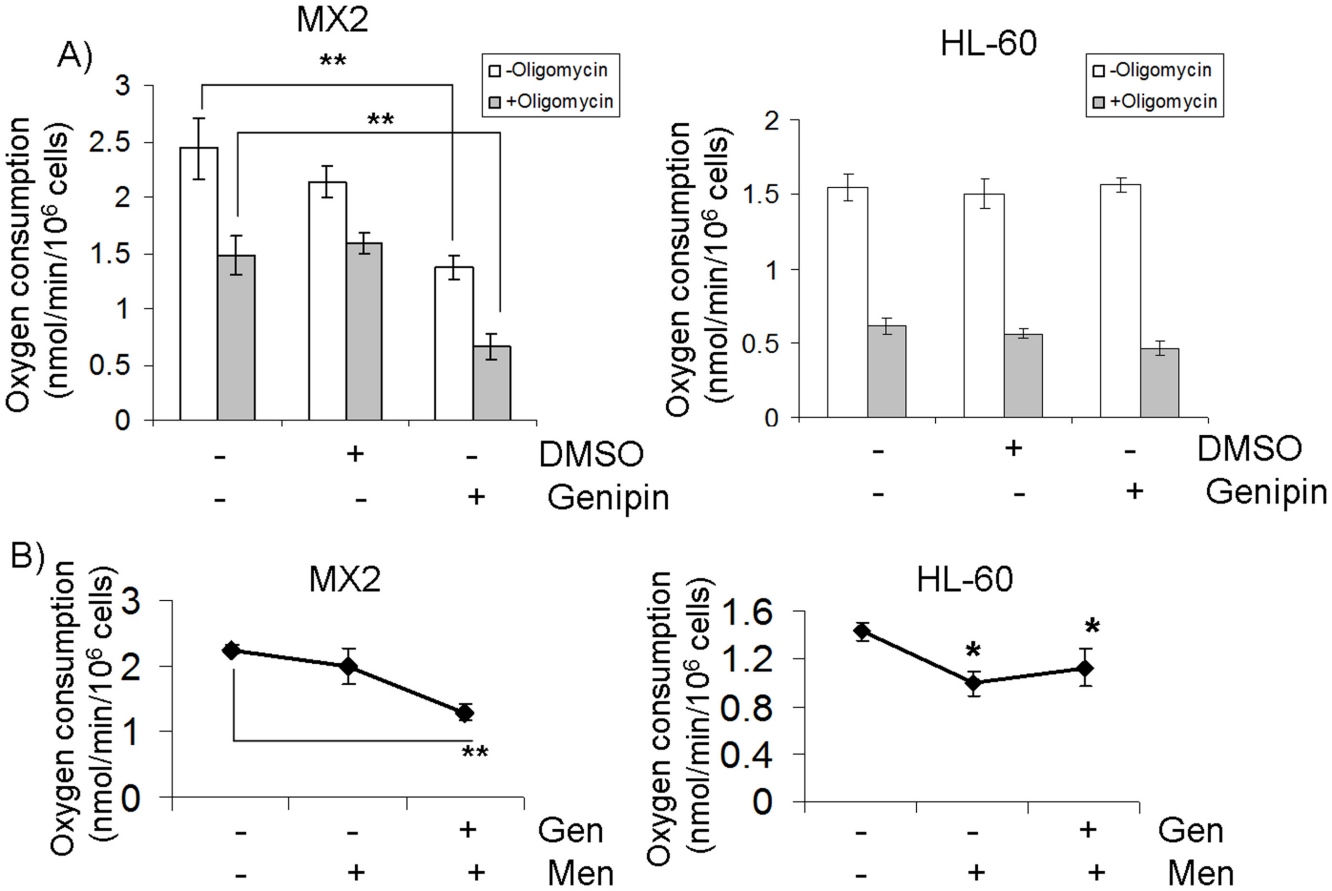
Genipin decreases cellular oxygen consumption in drug-resistant MX2 but not drug-sensitive HL-60 cells during oligomycin-induced state 4 respiration. A) Oxygen consumption measurements in cells exposed to 20 µM genipin 24 h exposure. Oxygen consumption measurements were performed following a 30 min incubation in reaction medium in the absence (white bars) or presence (grey bars) of oligomycin. 1-way ANOVA with a post-hoc Tukey's test, n = 6, **p<0.01. Treated MX2 or HL-60 cells were only compared with their corresponding control mean values. B) Oxygen consumption measurements in cells exposed to genipin (20 µM) and/or menadione (20 µM) for 24 h. 1-way ANOVA with a post-hoc Tukey's test, n = 4, *p<0.05,

Despite the glycolytic phenotype of cancer cells, the enzymatic machinery required for aerobic respiration frequently remains intact [44]. Maintenance of functional mitochondria is crucial for the cancer cell, *e.g.*, the Krebs cycle enzymes provide anabolic precursors for rapidly dividing cells [6]. Our group has shown previously that mitochondria in drug-resistant leukemia cells are uncoupled and that this is associated with decreased cellular ROS levels [10]. Thus, we hypothesized that the sensitizing effect of genipin was due to its ability to prevent UCP2-mediated uncoupling of the *Δψ*_m_. As shown in **Figure 3A**, the basal rate of oxygen consumption was higher in the MX2 when compared to HL-60 cells (∼54% greater, p<0.05). This indicates that the mitochondria in MX2 cells are more metabolically active than HL-60 mitochondria. Treatment of HL-60 and MX2 cells with oligomycin decreased respiration by ∼60% and ∼38% respectively (**Figure 3A**). This indicates that ∼62% of the resting cellular O_2_ consumption in the MX2 cells is due to uncoupled respiration (*e.g.* non-ATP producing activities). Sharp deviations in mitochondrial metabolism often accompany the acquisition of aggressive cancer phenotypes [6]. Indeed, targeted disruption of mitochondrial metabolism has been described as a potential cancer therapy [2]. Pre-treatment with genipin significantly diminished basal respiration in the MX2 cells (**Figure 3A**). These effects were not seen in the HL-60 cells. Genipin induced a ∼37% decrease in the respiratory rate revealing that UCP2 is quantitatively important in maintaining an uncoupled mitochondrial phenotype even under standard incubation conditions. In comparison, the CHO-UCP2 cells experienced a ∼22% decline in OCR following treatment with 50 µM genipin (**Figure 1C**). The discrepancy between the CHO and MX2 cells is most likely due to the differences in dose and the time of exposure between the two separate cells lines. This observation indicates overall that UCP2 is a major contributor to MX2 cellular bioenergetics. Moreover, following the addition of genipin to oligomycin-treated MX2 cells, the respiration rate was identical to that of the HL-60 cells (**Figure 3A**). This indicates that UCP2 was entirely responsible for the increased respiration of MX2 cells. Several studies have shown that genipin specifically inhibits UCP2-mediated uncoupling [34], [45]. For instance, Zhang *et al* illustrated that proton leak is not affected by genipin in UCP2^−/−^ cells and in tissue that does not express UCP2 [35]. We observed similar trends in the CHO cells exposed to genipin (**Figure 1C**). Hence, genipin completely inhibits the increased cellular respiration in the MX2 cells, while it has no effect on the HL-60 parent cells.

UCP2 is known to be a negative regulator of mitochondrial ROS production, a property attributed to its mild uncoupling activity. In two elegant studies, Echtay *et al* provided convincing evidence that UCP2-mediated proton conductance is maximally activated by oxidative stress in the mitochondria [27], [46]. This type of “inducible” proton leak has been described as an important safeguard in preventing increases in mitochondrial ROS emission. Thus, we tested if ROS treatment could increase proton leak in the MX2 cells. Menadione treatment did not alter oxygen consumption in the MX2 cells (**Figure 3B**). These data indicate that ROS did not cause further increases in proton leak dependent respiration. However, in the HL-60 cells, menadione treatment significantly diminished oxygen consumption, perhaps due to the diminished ability of these cells to control cellular ROS levels (**Figure 3B**). Mitochondrial metabolism (*e.g.*, citric acid cycle enzymes and electron transport chain proteins) is a major target for ROS toxicity. Thus, the decrease in oxygen consumption observed in the HL-60 cells exposed to menadione indicates these cells have a lower capacity to maintain tolerable levels of cellular ROS in the presence of cytotoxic agents. The above data establish that UCP2 prevents ROS-induced impairments in mitochondrial metabolism. In other words, the low abundance of UCP2 in the HL-60 cells may mean that they are unable to detoxify the ROS. In the MX2 cells, our results support the conclusion that UCP2 is already maximally activated. Indeed, in two separate experiments (**Figure 3A and 3B**) genipin diminished oxygen consumption by ∼37% in cells under standard incubation conditions. Furthermore, nonphosphorylating respiration accounted for ∼62% of the O_2_ consumption in the MX2 cells and genipin inhibited over half of this respiration, resulting in the normalization of non-phosphorylating respiration (*i.e.*, to HL-60 values). Hence, UCP2 accounts for a large fraction of the nonphosphorylating respiration in MX2 cells. Thus, the unique metabolic profile of the drug-resistant cancer cells appears to include enhanced UCP2-mediated mitochondrial uncoupling to avoid the cytotoxic effects of drug- and mitochondrial-borne ROS.

### Treatment with genipin enhances drug-induced ROS production

**Figure 4:**
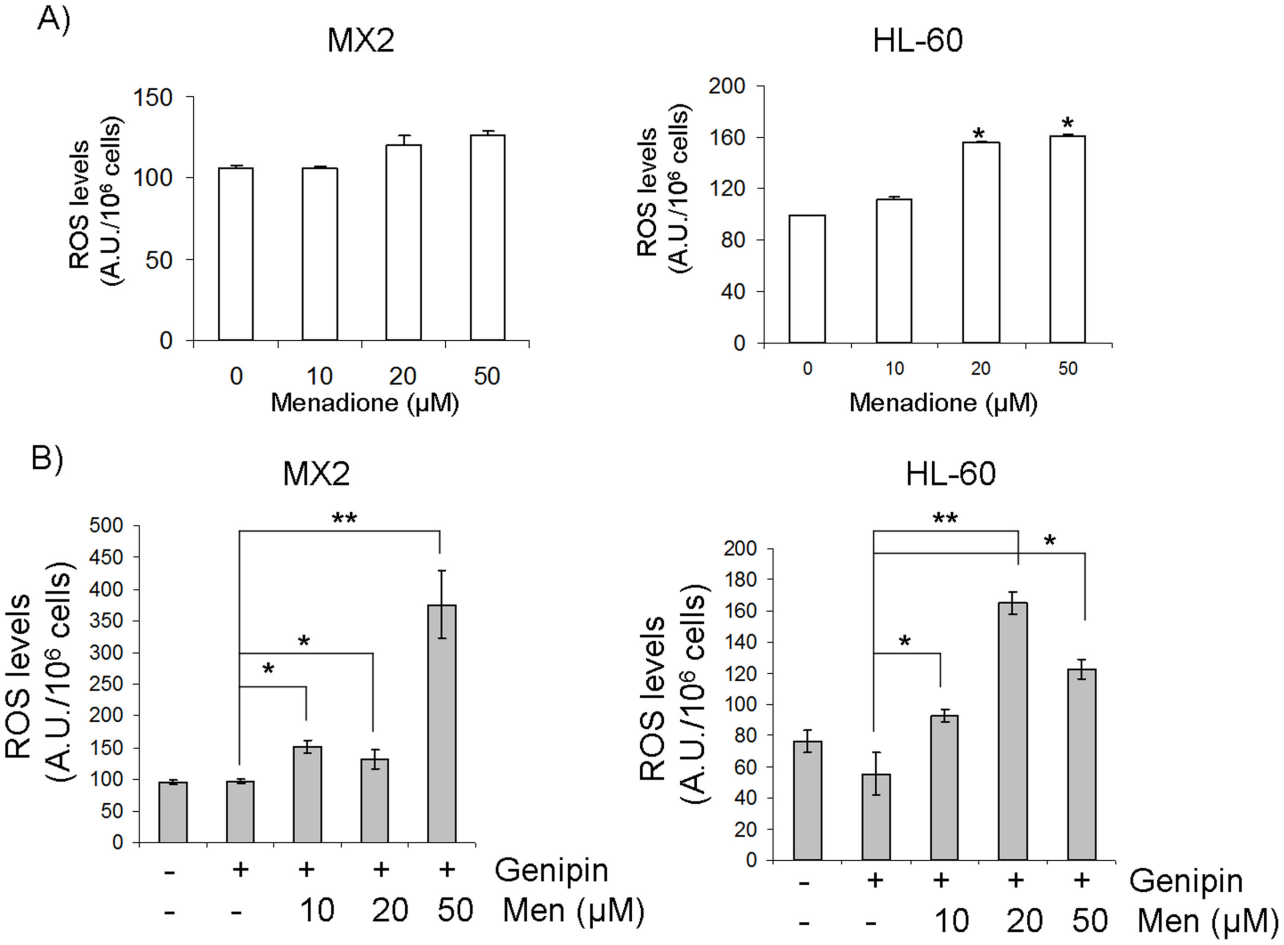
Genipin treatment increases menadione-mediated ROS levels in drug-resistant cells. ROS levels were assessed in intact HL-60 and MX2 cells using DCFH-DA. A) ROS levels in cells exposed to solely menadione (0–50 µM) for 24 h. 1-way ANOVA with a post-hoc Tukey's test, n = 5, *p<0.05. B) ROS levels in cells exposed to 0 or 20 µM genipin and menadione (0–50 µM) for 24 h. 1-way ANOVA with a post-hoc Tukey's test, n = 5, * p<0.05, **p<0.01.

The above data indicate that the inhibition of UCP2 by genipin results in the induction of drug-induced cell death in multidrug resistant MX2 cells. Since UCP2 inhibition sensitizes cells to ROS-producing agents, we then tested if genipin treatment enhanced drug-induced ROS formation. HL-60 cells treated with menadione alone produced far more ROS than their drug-resistant counterpart (**Figure 4A**). This observation is consistent with the enhanced ROS handling capacity of drug-resistant cells. However, the co-treatment of MX2 cells with genipin and menadione increased ROS levels. Indeed, exposure of genipin-treated MX2 cells to different amounts of menadione resulted in increased ROS levels (**Figure 4B**). At 50 µM menadione, there was a sharp increase in ROS levels in the genipin-exposed MX2 cells. Only moderate increases in ROS production were observed in the HL-60 cells treated with menadione and genipin (**Figure 4B**). Treatment with genipin alone in MX2 cells did not increase ROS levels (**Figure 4B**). Thus, genipin renders the MX2 cells more sensitive to treatment with ROS producing agents by interfering with proton leak.

**Figure 5:**
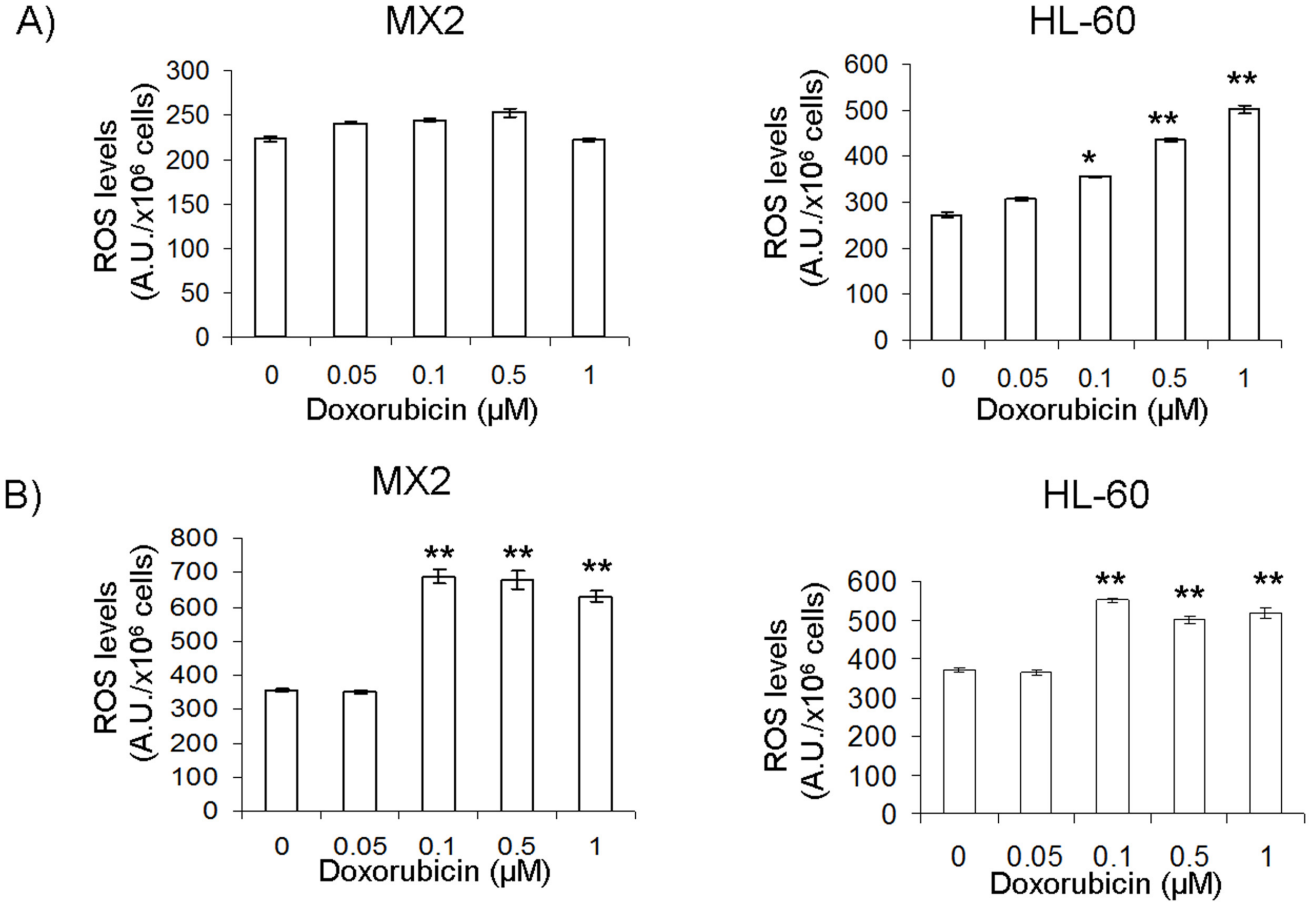
Genipin treatment enhances the production of ROS by doxorubicin. ROS levels were assessed in intact HL-60 and MX2 cells using DCFH-DA. A) ROS levels in HL-60 cells and MX2 cells exposed to doxorubicin (0–1 µM) for 24 h. 1-way ANOVA with a post-hoc Tukey's test, n = 5, *p<0.05, **p<0.01. B) ROS levels in HL-60 cells and MX2 cells exposed to 20 µM genipin and doxorubicin (0–1 µM) for 24 h. 1-way ANOVA with a post-hoc Tukey's test, n = 5, *p<0.05, **p<0.01.

Similar observations were made with doxorubicin. Doxorubicin is classically referred to as a topoisomerase II inhibitor [47]. However, other mechanisms of toxicity, such as ROS production, have been suggested [48]. Treatment with doxorubicin alone resulted in a dose-dependent increase in ROS levels in HL-60 cells (**Figure 5A**). In contrast, no increases in ROS were observed in the MX2 cells even at 1 µM. However, when genipin was included with the doxorubicin, increases in ROS levels were observed in the MX2 cells (**Figure 5B**). Indeed, exposure of genipin-loaded cells to concentrations of doxorubicin as low as 0.1 µM resulted in a sharp increase in ROS levels. Although doxorubicin is widely used as an anticancer agent, its mechanism of action is not fully understood. In this study, we show that doxorubicin can increase ROS levels in the MX2 cells upon co-treatment with genipin. It is quite possible that doxorubicin toxicity targets the mitochondria. The anthracyclins are quinone molecules, like menadione, which are capable of catalyzing the singlet electron reduction of diatomic oxygen to superoxide. Superoxide, at high enough concentrations, interferes with the proper functioning of the mitochondria. However, in this study the production of ROS by doxorubicin and menadione in MX2 cells required genipin treatment. It is entirely possible that the drug-resistant cells cope with the inhibitory effects of doxorubicin and quinones by maintaining mitochondria in an uncoupled state limiting ROS formation.

Our findings indicate that the natural product genipin can render drug-resistant cancer cells more sensitive to ROS-producing agents. The clinical application of genipin as a potential drug-sensitizing agent requires further investigation. Indeed, the drug-sensitizing effects of genipin were examined herein with commonly studied lines of cancer cells. Hence, to provide more clarity on the potential role of genipin in cancer treatment, comprehensive *in vivo* analyses are now required (*e.g.*, animal studies). However, this study provides some important preliminary insights into the potential use of genipin in cancer treatment. In this study, the sensitizing effect of genipin was attributed its interaction with UCP2, a protein abundantly expressed in the MX2 cell mitochondria. The specificity of genipin for UCP2 was confirmed with CHO cells stably expressing UCP2. HL-60 cells were unresponsive to genipin treatment which is most likely attributed to the low amounts of UCP2 expressed in these cells. The specific inhibitory action of genipin was confirmed using CHO cells either expressing or not expressing UCP2. Thus, our findings are consistent with a specific effect of genipin on UCP2. UCP2 is expressed in a number of normal somatic cells (splenocytes, pancreatic β cells, thymus cells, and various other immune-type cells) [26]. The amount expressed in drug-resistant cancer cells greatly exceeds the protein levels in non-cancerous cells and drug-sensitive cancer cells. Low UCP2 protein levels are maintained by tight translational control and rapid degradation [49]. Curiously, glutamine has been implicated in the induction of UCP2 mRNA translation [50] and drug-resistant cancer cells have enhanced glutamine uptake and metabolism, a process mediated by Myc [51]. Thus, it is possible that glutamine dependence plays a part in enhancing UCP2 protein expression in drug-resistant cells. It would be interesting to determine if UCP2 can be induced by Myc. In this study, the mechanism for the UCP2-mediated reduction in ROS stems from its uncoupling activity. Indeed, in MX2 cells UCP2 was responsible for over a third of resting cellular respiration, and approximately 60% of state 4 respiration. Other ROS detoxification mechanisms were enhanced in the MX2 cells however UCP2 displayed the greatest increase in abundance indicating that uncoupling plays a key role in controlling ROS levels. Further research is required to specifically address the possibility that UCP2 could be targeted by cell permeable compounds, such as genipin, to augment the effectiveness of cytotoxic strategies in cancer treatment.

## Materials and Methods

### Cell lines and treatment

The drug-sensitive and drug-resistant human acute promyelocytic leukemia (APL) suspension cell lines HL-60 and HL-60/MX2 (MX2) were purchased from the American Type Culture Collection on May 6 2009 (ATCC, Manassas, Virginia). Selection for sensitivity and resistance to chemotherapeutics and authentication were performed by ATCC prior to shipping. Upon receiving the cells were propagated and prepared for cryogenic storage according to the instructions provided by ATCC. Cells were routinely cultured in Dulbecco's Modified Eagle Medium (DMEM) supplemented with 20% (v/v) fetal bovine serum (FBS), 4 mM L-glutamine, 25 mM dextrose, 1 mM pyruvate, and 1% (v/v) antibiotics/antimycotics. Cells were cultured up to 15 passages and then discarded. Cells were provided with fresh media every 48 h. For experimentation, cells were grown to 70% confluency and then provided fresh media containing genipin (0–500 µM), menadione (0–100 µM), doxorubicin (0–10 µM), or epirubicin (0–10 µM). Cells were exposed to the different conditions for 24 h. Cells were then isolated and treated accordingly for each assay.

CHO cells stably transfected with either pcDNA3.1 vector containing UCP2 (CHO-UCP2) or empty vector (CHO-EV) were a gift from Dr. Frédéric Bouillaud [50]. CHO cells were routinely maintained in Matrigel-coated T75 cm^2^ flasks in DMEM containing 20% FBS and 2% antibiotics/antimycotics. Media was changed every two days and cells were passaged every four days. For the immunodetection of UCP2, cells were grown to confluency in Matrigel-coated 6-cm^2^ dishes and then lysed on ice with RIPA buffer (25 mM Tris-HCl pH 7.6, 150 mM NaCl, 1% NP-40, 1% sodium deoxycholate, 0.1% SDS, 1 mM NaF, 1 mM Na_3_VO_4_, 1 mM PMSF, and protease inhibitors). Cells were scraped from the dish and then prepared for immunoblot. Protein content was determined using the BCA assay.

### Cell Viability and Survival Assays

Cell survival following genipin, menadione, doxorubicin, or epirubicin exposure was ascertained using cell viability and Propidium Iodiode (PI) assays. For viability assays, the Trypan Blue exclusion method was employed [52]. Briefly, an aliquot of the cell suspension was diluted in Trypan Blue solution (Invitrogen, Burlington, Ontario) and cell viability was determined using the Countess Cell Counter according to the manufacturer's instructions (Invitrogen, Burlington, Ontario). For PI assays, cells were washed three times with washing buffer (PBS +10 mM dextrose) and then incubated for 10 min in PI (10 µg/mL in PBS). PI solution was removed and cells were washed twice. Cells were then placed in a 96-welled plate and read at an excitation wavelength of 530 nm and an emission wavelength of 615 nm (Biotek FLX-800, Fisher). PI results were normalized to protein content using the Bradford assay. PI (1 mg/mL) was purchased from Sigma (St Louis, Missouri).

### C2C12 myoblast culture

C2C12 myoblasts were routinely cultured in Matrigel coated 6-cm^2^ dishes in DMEM consisting of 25 mmol/L dextrose, 4 mmol/L L-glutamine, 20% (v/v) fetal bovine serum, and 1% (v/v) antibiotics/antimycotics. To assess the toxicity of genipin against a non-cancer cell line, C2C12 cells were seeded at 10,000 cells/mL in a final volume of 200 µL in Matrigel coated 96-welled dishes. Cells were grown to confluency and then treated with genipin (0–500 µM) for 24 h. The cell monolayer was then washed with PBS and treated with PI assay medium as described above. Following several washings, the degree of PI fluorescence associated with the cell monolayer was determined. The assay was normalized to protein level using the Bradford assay (BioRad, Mississauga, Ontario, Canada).

### Impact of genipin on CHO cell bioenergetics

The Seahorse XF24 Extracellular Flux Analyzer (Seahorse Bioscience, North Billerica, MA) was employed to determine the impact of genipin on the mitochondrial bioenergetic characteristics of CHO cells. CHO-UCP2 and CHO-EV cells were seeded at 50,000 cells/mL and grown to confluency in Matrigel-coated 24-welled Seahorse plates. Analyses were conducted on 20 wells per plate on three separate plates for an n = 3. Mean values were then calculated from the OCR per well in each plate following correction to total amount of cellular protein per well. For experiments, cells were then incubated for 30 min at 37°C in HCO_3_-free DMEM containing 10 mM glucose, 4 mM L-glutamine, and 1 mM pyruvate (pH 7.0). Fluorimetric sensors enabled the sensitive *in situ* measurement of O_2_ consumption rate (OCR). Measurement of OCR was performed over 2 min in three measurement intervals to assess basal metabolic rate (one measurement interval includes 2 min mixing, 2 min incubation, and 2 min measurement steps). Following the determination of basal OCR, cells were exposed to genipin (0–50 µM) for 15 min and then OCR was assessed. Data were expressed as percentages of the CHO-EV respiration rates (OCR/mg of protein).

### Measurement of cellular ROS levels

Intracellular ROS levels were assayed using 2′, 7′-dichlorodihydrofluorescin diacetate (DCFH-DA; Invitrogen). Following exposure to genipin (20 µM), menadione (0–50 µM), or doxorubicin (0–1 µM) cells were counted to determine live cell concentration. Cells were then harvested by centrifugation at 250 g for 5 min and washed once with washing buffer. The cell pellet was then resuspended in DMEM containing 20 µmol/L DCFH-DA. Cell suspensions were then incubated for 30 min at 37°C under constant agitation. Following two washes with washing buffer the cells were resuspended in 1 mL of washing buffer. Fluorescence was recorded using a 96-welled plate reader operating at an excitation/emission wavelength of 485 nm/530 nm (Biotek FLX-800, Fisher). Mean fluorescence values of DCFH-DA-loaded cells were corrected by subtracting the autofluorescence background.

### Measurement of oxygen consumption in HL-60 and MX2 cells

Measurements were performed as described in [10]. Cells grown in the presence or absence of genipin (20 µM) or menadione (20 µM) were harvested, washed once with washing buffer and placed on ice prior to experimentation. Batches of 10×10^6^ cells were resuspended in 1 mL of reaction medium (106 mmol/L NaCl, 0.41 mmol/L MgCl_2_, 25 mmol/L Na_2_HPO_4_, 5 mM KCl, 10 mM dextrose, pH 7.0) and incubated for 30 min at 37°C. Oligomycin (10 µg/mL) was included in the reaction medium to determine the contribution of proton leak to oxygen consumption. The cell suspension was then placed in the temperature-regulated Clark-type electrode chamber (Oxytherm; Hansatech Instruments Ltd, Norfolk England). Oxygen consumption was measured for 5-15 min (until oxygen consumption ceased) at 37°C. The rate of oxygen consumption for each run was determined using Oxytherm Plus software.

### Mitochondrial isolation

Mitochondria were isolated using the protocol described in [53] with some minor modifications. Briefly, 100×10^6^ cells were harvested and washed with ice-cold washing buffer. Cells were then resuspended in a slightly hypotonic mitochondrial isolation buffer (250 mmol/L sucrose, 10 mmol/L Hepes, 1 mmol/L EDTA, protease inhibitor cocktail (Roche, Mississauga, Ontario, Canada), pH 7.4) and sonicated four times on ice for 10 seconds in 1 second bursts. After each sonication the cell suspension was placed on ice for 5 min. The hypotonic medium provided an enriched mitochondrial preparation as opposed the KCl-based isotonic mitochondrial isolation media commonly employed. Cell suspensions were then subjected to differential centrifugation to isolate the mitochondria. To isolate mitochondria, the cell suspensions were centrifuged at 250×g and 850×g for 10 min at 4°C to remove whole cells and nuclei. The supernatant was then centrifuged at 12,000×g for 30 min at 4°C to yield the mitochondrial pellet and a cytosolic fraction. Protein content was determined using the Bradford assay (Bio-Rad, Mississauga, Ontario, Canada).

#### Immunoblot analysis

Samples were diluted to 1 mg/mL in Laemmli buffer and 30 µg of protein was electrophoresed on a 12% isocratic SDS-gel. Concentrated spleen lysate, which is known to express UCP2, served as a control (100 µg of protein was loaded and electrophoresed). Transfers to nitrocellulose membranes were performed at room temperature at a voltage of 100V. The transfer buffer consisted of 1% (v/v) SDS to ensure complete transfer of proteins. Transfer efficiency was tested by Ponceau S staining of membranes and Coomassie R-250 staining the gels. The membranes were blocked and probed for 1–24 h at 4°C with primary antibodies directed against UCP2 (anti-N19 and anti-C20, 1/2000 dilution, Santa Cruz), Mn-dependent superoxide dismutase (MnSOD, 1/2000 dilution, Santa Cruz), glucose-6-phosphate dehydrogenase (G6PDH, 1/3000 dilution, Santa Cruz), and glutathione peroxidase (GPx, 1/1000 dilution, Abcam). Various primary antibody dilutions were used to optimize detection (1/200-1/5000). NADP-dependent isocitrate dehydrogenase (ICDH, 1/2000, Abcam) was used as a loading control for the cytosol and mitochondrial fractions (the ICDH antibody reacts with both the cytosol and mitochondrial isoforms of ICDH). Succinate dehydrogenase (SDH, 1/2000, Santa Cruz) served as loading control for the CHO cells. Membranes were then incubated for 1 h at room temperature with the requisite horseradish peroxidase-conjugated secondary antibody (anti-rabbit, anti-mouse, or anti-goat, 1/2000, Santa Cruz). Blots were visualized using enhanced chemiluminescent substrate (ECL kit, Thermo Scientific).

#### Statistical Analysis

One-way ANOVA with a post hoc Tukey's test and Kruskall-Wallis with a post-hoc Mann-Whitney non-parametric test (Statview software, SAS Institute Inc., USA) were used to assess statistical differences. All p values were generated from the post-hoc tests. Results are expressed as mean value +/− standard deviation.

## Supporting Information

Figure S1 A) Determination of cell death in MX2 cells exposed to menadione (0–100 µmol/L). Following a 24 h exposure to menadione, amount of cell death was determined using the PI assay. Data were expressed as a percent of the control. 1-way ANOVA with a post-hoc Tukeys test, n = 5, **p<0.01. All treated means were compared to the control mean. B) Toxicity of genipin towards noncancer cells. Confluent C2C12 cells were exposed to genipin (0–500 µM) for 24 h and the degree of cell death was determined by PI assay. 1-way ANOVA with a post-hoc Tukeys test, n = 4, *p<0.05. All treated means were compared to the control mean.
(0.02 MB PDF)
