# Supplementary figures and images for: Genipin-Induced Inhibition of Uncoupling Protein-2 Sensitizes Drug-Resistant Cancer Cells to Cytotoxic Agents
Source: PLoS One. 2010 Oct 13;5(10):e13289. doi: 10.1371/journal.pone.0013289 (PMC2953501; doi:10.1371/journal.pone.0013289)

Supplemental Figure 1

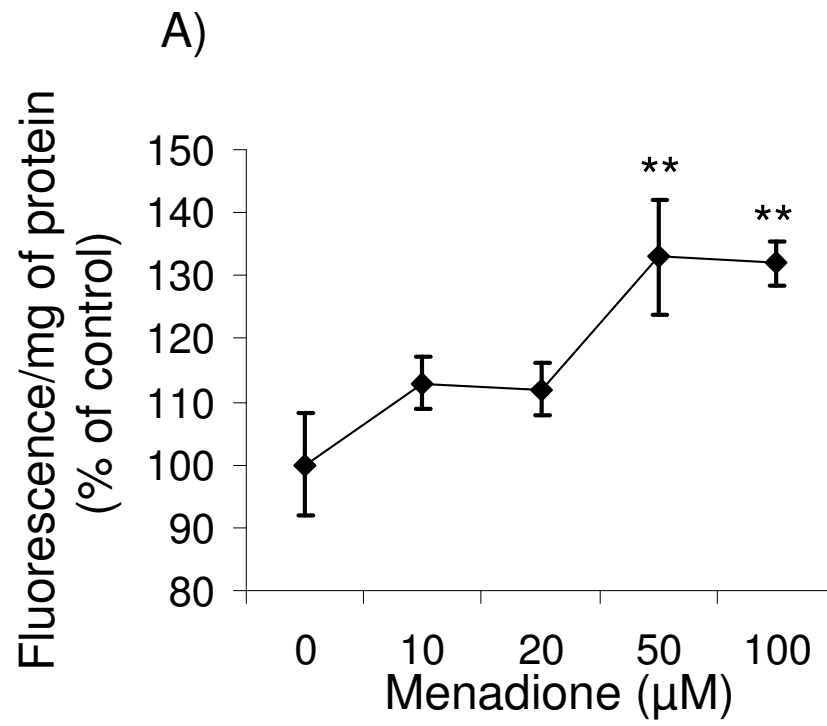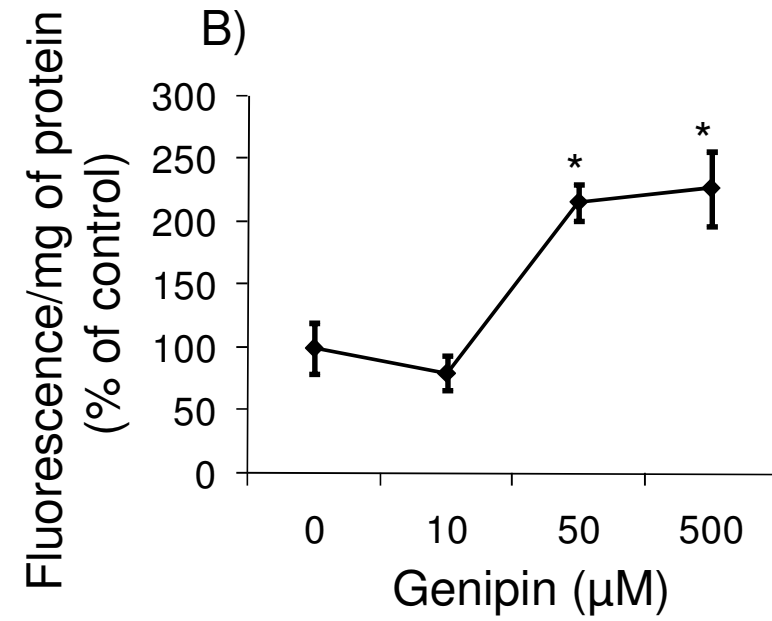

Supplement: Figure S1 — A) Determination of cell death in MX2 cells exposed to menadione (0–100 µmol/L). Following a 24 h exposure to menadione, amount of cell death was determined using the PI assay. Data were expressed as a percent of the control. 1-way ANOVA with a post-hoc Tukeys test, n = 5, **p<0.01. All treated means were compared to the control mean. B) Toxicity of genipin towards noncancer cells. Confluent C2C12 cells were exposed to genipin (0–500 µM) for 24 h and the degree of cell death was determined by PI assay. 1-way ANOVA with a post-hoc Tukeys test, n = 4, *p<0.05. All treated means were compared to the control mean. (0.02 MB PDF) [file pone.0013289.s001.pdf]
